# Risk factors for severe and fatal childhood unintentional injury: a systematic review protocol

**DOI:** 10.1186/s13643-024-02612-2

**Published:** 2024-07-24

**Authors:** Emilie Beaulieu, Norma Maria Perez Herrera, Amélie Boutin

**Affiliations:** 1 Reproduction, Mother and Youth Health Unit, CHU de Québec — Université Laval Research Center, 2705 Boulevard Laurier, Quebec City, QC G1V 4G2 Canada; 2 Population Health and Optimal Health Practices Unit, CHU de Québec — Université Laval Research Center, 1050 Chemin Ste-Foy, Quebec City, QC Canada; 3 Department of Pediatrics, Université Laval, 2705 Boulevard Laurier, R1742, Quebec City, QC G1V 4G2 Canada

**Keywords:** Childhood injury, Risk factors, Injury prevention, Injury determinants

## Abstract

**Background:**

Unintentional injuries are a leading cause of death among children aged 1–19 years worldwide. Systematic reviews assessing various risk factors for different childhood injuries have been published previously. However, most of the related literature does not distinguish minor from severe or fatal injuries. This study aims to describe and summarize the current knowledge on the determinants of severe and fatal childhood unintentional injuries and to discuss the differences between risk factors for all injuries (including minor injuries) and severe and fatal injuries. The study also aims to quantify the reduction in childhood injuries associated with a reduction in exposure to some of the identified risk factors in the Canadian population.

**Methods:**

A systematic review and meta-analysis will be conducted by searching MEDLINE, Embase, CINAHL, and Web of Science. Observational and experimental cohort studies assessing children and adolescents aged ≤ 19 years old and determinants of severe and fatal unintentional injury, such as personal behaviors, family and environmental characteristics, and socioeconomic and geographic context, will be eligible. The main outcome will be a composite of any severe or fatal unintentional injuries (including burns, drowning, transport-related injuries, and falls). Any severity measurement scale will be accepted as long as severe cases require at least one hospital admission. Two authors will independently screen for inclusion, extract data, and assess the quality of the data using the Cochrane ROBINS-E tool. Meta-analysis will be performed using random effects models. Subgroup analyses will examine age subgroups and high- vs low-income countries. Sensitivity analysis will be conducted after restricting analyses to studies with a low risk of bias. Attributable fractions will be computed to assess the burden of identified risk factors in the Canadian population.

**Discussion:**

Given the numerous determinants of childhood injuries and the challenges that may be involved in identifying which individuals should be prioritized for injury prevention efforts, this evidence may help to inform the identification of high-risk children and prevention interventions, considering the disproportionate consequences of severe and fatal injuries. This evidence may also help pediatric healthcare providers prioritize counseling messaging.

**Systematic review registration:**

PROSPERO CRD42023493322.

**Supplementary Information:**

The online version contains supplementary material available at 10.1186/s13643-024-02612-2.

## Background

Unintentional injuries, including burns, drowning, transport-related injuries, and falls, are leading causes of death among children aged 1–19 years worldwide [1]. Many studies have provided useful descriptive portraits of such events over the years, including injury type, location, and distribution according to sex and age [2, 3]. To guide injury prevention programs, researchers have reviewed and synthesized the individual, family, and environmental determinants associated with child unintentional injuries [4–6]. Systematic reviews assessing various risk factors for different childhood injuries have been published previously [5, 7–9]. The important number of risk factors for unintentional injury in children reported in the literature illustrates the complex and multidisciplinary nature of injury prevention [10].

Most of the systematic reviews assessing child injury risk factors do not differentiate between minor and severe or fatal injuries [5, 7–9]. Identifying risk factors for severe and fatal unintentional injuries, given their disproportionate consequences compared to minor injuries, may help to prioritize prevention efforts. It may also help child health providers identify at-risk children through individual, family, environmental, and socioeconomic determinants to concentrate injury prevention counseling, in addition to universal messaging, on this population. This study aims to describe and summarize the current knowledge on the determinants of severe and fatal childhood unintentional injuries and to quantify the reduction in child injuries if exposure to some of the identified risk factors was reduced in the Canadian population. The study also aims to discuss the differences between risk factors for all injuries (including minor injuries) and severe and fatal injuries.

## Methods

This systematic review will be conducted according to Cochrane guidelines, and the results will be reported according to the Preferred Reporting Items for Systematic Reviews and Meta-Analyses (PRISMA) statement and the Meta-analysis Of Observational Studies in Epidemiology (MOOSE) reporting guidelines [11]. The protocol is reported according to the Preferred Reporting Items for Systematic Reviews and Meta-Analyses-Protocols (PRISMA-P) statement (Appendix 1) and has been registered in the International Prospective Register of Systematic Reviews (PROSPERO no. CRD42023493322).

### Eligibility criteria

Studies including primarily children and adolescents aged ≤ 19 years old assessing any determinants of severe or fatal unintentional injury, such as personal behaviors, family and environmental characteristics, and socioeconomic and geographic context, will be eligible [12]. Severe injuries will include any injury that required at least one hospital admission or abbreviated injury scale (AIS) ≥ 3 [13]. Studies assessing injuries that necessitated a clinic or emergency room visit only or that did not differentiate severe and fatal injuries from minor injuries will be excluded. Studies addressing gunshot wounds and poisoning will not be included, given that a considerable proportion of these events may be intentional with different sets of risk factors. Observational studies (retrospective and prospective cohorts, longitudinal studies, epidemiological studies, case controls) and experimental cohorts will be included. Studies assessing the effectiveness of any injury prevention interventions will not be included. Studies providing only descriptive statistics (frequencies) of the characteristics of injuries (i.e., time of injury, location, etc.) and studies for which the measure of risk was not between a determinant and the injury outcome will not be eligible. There will be no publication date restrictions, and languages other than French, English, or Spanish will be excluded.

### Information sources

The following databases will be searched systematically: MEDLINE (OVID), Embase (Elsevier), CINAHL (EBSCO), and Web of Science (including Science Citation Index Expanded [SCI-EXPANDED], Social Sciences Citation Index [ESCI], Arts & Humanities Citation Index [AHCI], and Emerging Sources Citation Index) from their inception to a maximum of 6 months prior to submission for publication. References of identified studies and prior systematic reviews of determinants of childhood injuries will be manually screened to find additional articles. Google Scholar and ProQuest will be used to search the gray literature.

### Search strategy

Search strategies will be developed in collaboration with a specialized librarian using an iterative process. The MEDLINE database will be searched first, and the approved search strategy will be adapted and applied to Embase, CINAHL, and Web of Science. The search strategy will use a combination of descriptors (e.g., MeSH) and keywords under the following themes: children and youth, severe and fatal injury, risk factors, and observational studies (Appendix 2).

### Study selection

Search results (including titles and abstracts) will be imported into Covidence systematic review software (Veritas Health Innovation, Melbourne, Australia) [14], and duplicates will be removed. Two independent reviewers will screen titles and abstracts using the criteria above. Studies that both reviewers agree to exclude will be disregarded by default. The full texts of the remaining articles will be evaluated by two independent reviewers to determine eligibility for final inclusion. Authors of studies with insufficient or unclear results will be contacted, but such studies will be excluded if no precision is possible after two contact attempts. Reasons for excluding studies at the full-text screening stage will be documented. In case of disagreement between the two reviewers despite discussions, a third reviewer will be called upon to adjudicate. Finally, a flow diagram summarizing the selection process will be created.

### Data collection

Two review authors will independently extract data from the full texts of the included studies using a data extraction form. Two review authors will pilot the data extraction form in a random sample of three studies to assess the consistency of the extracted data and will revise the form as needed. The extracted data will be cross-checked, and any discrepancies will be resolved through discussion or via a third author if no consensus can be reached. If some information or data are missing or uncertain, the study authors will be contacted to obtain additional information or data.

Extracted items will include study characteristics (first author, year of publication, country of study population, data sources and period, study design), population characteristics (sample size, age range, injury types, mechanism and severity), characteristics or risk factors (definition, any determinant will be accepted), and outcome characteristics (definition of severe/fatal unintentional injury in the study, including injury severity scales if available). We will extract data allowing the calculation of crude associations (e.g., mean and standard deviation or median and interquartile range for continuous variables and counts with favorable vs unfavorable outcomes for binary outcomes) or the strength of association in multivariate models (along with type of measure of association and list of variables included in the model).

All determinants, including clinical, behavioral, family, and environmental characteristics and socioeconomic and geographic contexts, will be considered for extraction.

The main outcome will be a composite of any severe or fatal unintentional injuries (including burns, drowning, transport-related injuries, and falls). Any severity measurement scale will be accepted as long as severe cases require at least one hospital admission. Secondary outcomes will include specific types of unintentional injuries.

### Risk of bias in individual studies

Two review authors will independently assess each included study for risk of bias using the “Risk Of Bias In Non-randomized Studies-of Exposure (ROBINS-E)” tool [15]. This tool grades 7 domains of bias as low, moderate, high or very high risk of bias, or no information. The domains include confounding, measurement of the exposures, selection of participants into the study, postexposure interventions, missing data, measurement of outcomes, and selection of the reported result. The “risk-of-bias” tool will be pilot-tested first with a random sample of three studies to assess the consistency of risk judgment between the two reviewers and the need to adapt the tool as needed. If experimental studies are included, the revised Cochrane risk-of-bias tool (RoB 2), which includes five domains of bias, will be used [16]. If at least one domain is considered to have a high risk or very high risk of bias, the overall assessment will categorize the study as having a high risk of bias. Otherwise, if at least one domain is considered “moderate,” the study will be classified as having an overall moderate risk of bias. Otherwise, if one domain is considered to have an “unclear” risk of bias, the study will be categorized as having an overall “unclear” risk of bias. Studies with all domains considered to be at low risk of bias will be categorized as “low risk.”

Risk-of-bias assessments according to each domain of bias and overall risk of bias will be synthesized graphically, separately for experimental and observational cohort studies and case–control studies, and reported in the summary of findings table.

### Data synthesis

#### Quantitative analyses

Meta-analyses will be performed only if at least three different eligible studies can be included in a specific analysis. For continuous variables, standardized mean differences between individuals with favorable vs unfavorable outcomes and 95% CIs will be pooled using the inverse variance method with random effects models at the study level to account for expected heterogeneity. For binary variables, cumulative incidences will be reported, and risk ratios will be pooled using Mantel–Haenszel methods with random effects models. Measures of associations between determinants and outcomes obtained from multivariate models will be pooled using the inverse variance method with random effects models. In the case of high heterogeneity of confounding factors taken into consideration, if possible, we will conduct subgroup analyses with greater homogeneity. Odds ratios and risk ratios will be pooled together, while hazard ratios will be pooled separately. The data from cohorts and case-control studies will be pooled separately.

#### Subgroup and sensitivity analyses

Sensitivity analyses will be conducted after restricting analyses to studies with a low risk of bias. If at least three studies are available, subgroup analyses will examine age subgroups (e.g., studies of toddlers, studies of teenagers, etc.) and high- vs low-income countries. Subgroup differences will be assessed with *χ*^*2*^ test.

For all analyses, we will assess heterogeneity using the *I*^2^ statistic. We will consider a type I error of 5%. Analyses will be conducted using Review Manager 5.

#### Attributable fractions

To assess the burden of determinants of severe and fatal unintentional injuries in the Canadian population, we will compute the attributable fractions when possible. The prevalence of risk factors will be estimated using data from Statistics Canada or through a review of the literature. The adjusted relative risk will be used for the calculation of population-attributable fractions.

#### Qualitative synthesis

In case insufficient data are available to conduct a meta-analysis, we will report crude data and measures of associations for individual studies.

### Meta-bias

Publication bias will be explored visually using funnel plots.

We will also report on the range of determinants reported in the literature to identify domains of determinants potentially underreported. We will use the Cochrane equity acronym “PROGRESS-Plus” (e.g., place of residence, race/ethnicity/culture/language, occupation, gender/sex, religion, education, socioeconomic status, and social capital) [17] to report equity-related determinants.

## Discussion

The findings of this review will provide evidence on risk factors for severe and fatal unintentional injuries among children. Given the numerous determinants of child injuries and the challenges that may be involved in identifying which individuals should be prioritized for injury prevention efforts, this evidence may help to inform the identification of high-risk children and prevention interventions, considering the disproportionate consequences of severe and fatal injuries. This evidence may also help pediatric healthcare providers prioritize counseling messaging.

We expect that injury types, determinants, and risk characterization of the relationship between determinants and injury may be too heterogeneous to conduct meta-analyses. Pooling of adjusted measures of association may also prove challenging if there is high heterogeneity in the variables included in multivariate models. We anticipate that many studies will be excluded because of the uncharacterized severity of the injury. However, several systematic reviews have assessed risk factors for childhood injury of all severities.

Our review will be based on an exhaustive search strategy and rigorous methodology. This study will therefore provide a synthesis of the evidence of the determinants of severe and fatal unintentional injuries in children, allowing us to identify optimal targets for preventive interventions and areas of scarce evidence. Thus, identifying gaps and avenues for future research to develop and improve preventive approaches will be possible.

### Supplementary Information

Supplementary Material 1: Appendix 1. PRISMA-P (Preferred Reporting Items for Systematic review and Meta-Analysis Protocols) 2015 checklist: recommended items to address in a systematic review protocol.

Supplementary Material 2: Appendix 2. Search strategy for MEDLINE (Ovid).

## Data Availability

Data sharing is not applicable to this article as no datasets were generated or analyzed during the current study.
