# Supplementary material for: Risk factors for severe and fatal childhood unintentional injury: a systematic review protocol
Source: Syst Rev. 2024 Jul 24;13:193. doi: 10.1186/s13643-024-02612-2 (PMC11267828; doi:10.1186/s13643-024-02612-2)
Supplement: Supplementary file 2 — Supplementary Material 2: Appendix 2. Search strategy for MEDLINE (Ovid). [file 13643_2024_2612_MOESM2_ESM.pdf]

Search Strategy for Medline (Ovid)

Concept 1: Child	
1	exp Child/ or Infant/ or Adolescent/ or Child hospitalized/ or Adolescent hospitalized/
2	(Child* or Infant* or pre-school* or preschool* or toddler* or Adolescen* or youth* or teenage* or teen or teens or "young people").ab,ti,kf.
3	1 or 2
Concept 2: Severe and fatal unintentional injury	
4	wounds and injuries/ep or accidental injuries/ or asphyxia/ or burns/ or drowning/ or near drowning/ or electric injuries/ or occupational injuries/ or reinjuries/ or accidents/ or accidental falls/ or accidents, home/ or accidents, occupational/ or accidents, traffic/
5	Death/ or Child Mortality/ or Hospital Mortality/ or Infant Mortality/ or Advanced Trauma Life Support Care/ or exp Trauma Severity Indices/
6	(sever* or serious or fatal* or death or mortal*).ab,ti,kf.
7	5 or 6
8	4 and 7
9	((injur* or burn* or accident* or drown*) adj2 (sever* or serious or fatal* or death or mortal* or hospital*)).ab,kf,ti.
10	or/8-9
11	Acute Kidney Injury.ab,kf,ti.
12	10 not 11
Concept 3: Risk factors	
13	Risk Factors/ or Protective Factors/ or sociodemographic factors/ or Socioeconomic Factors/ or exp social environment/ or Epidemiologic Factors/
14	((Risk adj2 factor*) or ((socioeconomic* or sociodemographic* or epidemiologic*) adj2 (factor* or status)) or ((Precipitating or protective) adj2 Factor*) or (social adj2 environment*) or (risk adj3 (injur* or hospitali#ation*))).ab,kf,ti.
15	13 or 14
Concept 4: Study types	
16	exp cohort studies/
17	cohort$.tw.
18	controlled clinical trial.pt.
19	epidemiologic methods/
20	limit 19 to yr=1966-1989
21	exp case-control studies/
22	(case$ and control$).tw.
23	or/16-18,20-22
24	**3 and 12 and 15 and 23**
